# Investigation of 89 candidate gene variants for effects on all-cause mortality following acute coronary syndrome

**DOI:** 10.1186/1471-2350-9-66

**Published:** 2008-07-12

**Authors:** Thomas M Morgan, Lan Xiao, Patrick Lyons, Bethany Kassebaum, Harlan M Krumholz, John A Spertus

**Affiliations:** 1 Department of Pediatrics, Vanderbilt University School of Medicine, Nashville, TN, USA; 2 Mid-America Heart Institute and University of Missouri-Kansas City, Kansas City, MO, USA; 3 Department of Pediatrics, Washington University School of Medicine, St. Louis, MO, USA; 4 Robert Wood Johnson Clinical Scholar Program and Department of Internal Medicine, Yale University School of Medicine, New Haven, CT, USA

## Abstract

**Background:**

Many candidate genes have been reported to be risk factors for acute coronary syndrome (ACS), but their impact on clinical prognosis following ACS is unknown.

**Methods:**

We examined the association of putative genetic risk factors with 3-year post-ACS mortality in 811 ACS survivors at university-affiliated hospitals in Kansas City, Missouri. Through a systematic literature search, we first identified genetic variants reported as susceptibility factors for atherosclerosis or ACS. Restricting our analysis to whites, so as to avoid confounding from racial admixture, we genotyped ACS cases for 89 genetic variants in 72 genes, and performed individual Kaplan-Meier survival analyses. We then performed Cox regression to create multivariate risk prediction models that further minimized potential confounding.

**Results:**

Of 89 variants tested, 16 were potentially associated with mortality (P < 0.1 for all), of which 6 were significantly associated (P < 0.05) with mortality following ACS. While these findings are not more than what would be expected by chance (P = 0.28), even after Bonferroni correction and adjustment for traditional cardiac risk factors, the *IRS1 *972Arg variant association (P = 0.001) retained borderline statistical significance (P < 0.1).

**Conclusion:**

With the possible exception of *IRS1*, we conclude that multiple candidate genes were not associated with post-ACS mortality in our patient cohort. Because of power limitations, the 16 gene variants with P values < 0.1 may warrant further study. Our data do not support the hypothesis that the remaining 73 genes have substantial, clinically significant association with mortality after an ACS.

## Background

Despite convincing evidence of heritable susceptibility to acute coronary syndromes (ACS), including unstable angina (UA), non-ST elevation myocardial infarction (NSTEMI), and ST-elevation myocardial infarction (STEMI),[1] most common genetic variants have yet to be validated conclusively as ACS risk factors. [2-4] We recently reported an attempt to validate putative cardiac risk factors,[5] all of which had been previously published in significant association with ACS or atherosclerosis, finding that only one pre-specified genetic risk genotype, homozygosity for the -455 A/G promoter variant in B-fibrinogen, could be replicated in our sample of 811 ACS cases and 650 controls. We concluded that our results did not support previous hypotheses generated by the candidate gene approach to ACS and that better study designs and unbiased screening approaches, such as whole genome association studies that have implicated 5 variants in the 9p21 region as ACS susceptibility factors,[6,7] would be needed.

However, it is not yet known if candidate gene variants, including those in the 9p21 region, will affect prognosis following ACS. In theory, genes associated with the development of clinically significant atherosclerosis may also have a strong impact on subsequent prognosis after an ACS event has occurred. In particular, those who have already suffered ACS are at very high risk for suffering a subsequent fatal or nonfatal cardiac event, due not only to post-ACS physiological vulnerability, but also to the genetic and environmental risk factor profiles that led to the initial cardiac event. Against this backdrop of heightened risk, individual genetic effects may be markedly potentiated. With so many individuals currently surviving ACS events worldwide, there is an urgent need to identify improved methods for risk-stratifying patients' post-ACS prognosis so that more aggressive efforts at secondary prevention or intracardiac defibrillator insertion can be considered. Moreover, the identification of clinically important genetic risks for an adverse outcome after an ACS can also identify new targets for potential intervention. Despite the importance of such investigations, few studies have examined the association of potential atherogenic risk factors on prognosis after an ACS. To stimulate this line of research, we have performed a longitudinal follow-up study to investigate the impact of 89 candidate gene variants on mortality following ACS.

## Methods

### Candidate genes

Our search strategy for putative risk variants, along with a list of references for each variant, has been described previously,[5] and further details about each gene variant, including flanking DNA sequences, are available upon request from the authors. Reports were included if they contained a claim of a significant positive association, with an investigator-reported P value < 0.05. Medline search terms included: "gene, genetic, polymorphism, myocardial infarction, atherosclerosis, coronary heart disease, and coronary artery disease." In addition to the gene variants contained in our initial report, we genotyped the 5 variants in the 9p21 region that have recently been implicated in the occurrence of myocardial infarction. Overall, we identified, and successfully genotyped, 89 variants in 72 genes (or gene regions, in the case of 9p21, given that a causative mechanism has not yet been identified).

### Study population and genotyping

Eight hundred eleven self-reported white patients of European ancestry with ACS were identified from a consecutive series of patients presenting to two Kansas City, MO hospitals (Mid-America Heart Institute and Truman Medical Center), from March 2001 through June 2003. Standard definitions were used to diagnose ACS patients with either myocardial infarction or unstable angina.[8,9] Individuals were monitored for incident deaths from any cause, as determined by periodic queries of the Social Security Administration Death Master File.[10] A minimum of 3 years of follow-up was available.

Genotyping was performed using the Sequenom MALDI-TOF (Matrix Assisted Laser Desorption-Ionization Time-of-Flight)[11,12] system on whole genome amplified DNA.[13,14] More extensive details of the cases and genotyping procedures have been recently described.[5]

### Statistical Analysis

Genotype distributions were examined for significant deviation (P < 0.05) from Hardy-Weinberg equilibrium. PHASE Version 2.1 was used to estimate haplotype frequencies for *ALOX5AP*.[15,16]

Initially, Kaplan-Meier survival analysis was performed for each variant (SAS 9.1, Research Triangle Park, NC). Cox regression models were then used to adjust positive associations for cardiac risk factors (age, sex, hypertension, congestive heart failure, diabetes), type of ACS (STEMI, NSTEMI, UA), and ACS treatments (aspirin and beta-blocker treatments provided in the first 24 hours, the use of angiography and revascularization, as well as quality of care indicators rendered at discharge (aspirin, beta-blocker, ACE inhibitor, and smoking cessation counseling)). We tested the proportional-hazards assumption for each covariate, by correlating the corresponding set of scaled Schoenfeld residuals with a suitable transformation of time based on the Kaplan-Meier estimate of the survival function. We applied the conservative Bonferroni correction in considering the overall statistical significance of results,[17] but we also simply compared the total number of all positive associations at the P < 0.05 level to the expected number by chance, in 50,000 simulations (Resampling Stats, Inc., College Park, MD). An unexpected surplus of positive associations would imply that some of the tested polymorphisms may be truly associated with ACS.

Our sample had 93% power to detect an association, by the log rank test (P < 0.05), for a hazard ratio of 2.5 or higher, given a frequent genotype (0.5), and 80% power to detect a hazard ratio of 3.3 or higher, given an infrequent genotype (0.1).[18] For weak effects (1.1–1.5 hazard ratio), power was limited, ranging from 5% (0.1 genotype frequency, 1.1 hazard) to 29% (0.5 genotype frequency, 1.5 hazard). Given that such genetic variants with modest effects may not reach the conventional statistical significance level of P < 0.05, we sought to explore the possibility that null results might be related to lack of power (type II error), by examining the characteristics of the overall P value distribution. A Q-Q plot was performed, and supplemented with a simulation-based analysis. Random P values follow the Beta distribution. Hence, we subtracted random numbers between 0 and 1 (Beta distribution) from the observed P values, computed mean (observed-random) differences across all genetic variants, repeated this procedure 50,000 times, and plotted frequency histograms of the resulting 50,000 mean differences (Resampling Stats, Inc). In the presence of multiple *bona fide *genetic risk factors, but suboptimal power, the average mean P value difference (observed-random) would be less than zero. We initially included all P values from the individual Kaplan-Meier log-rank tests for all 89 genetic variants, and then sequentially removed those with the lowest P values until the remaining subset converged to a difference equal to zero, as would be expected for random variables showing no evidence of association with ACS.

## Results

The clinical characteristics of the 811 cases are described in Table 1. The population of ACS cases included 308 (38%) STEMI, 284 (35%) NSTEMI, and 219 (27%) UA patients. A family history of coronary artery disease or myocardial infarction among first degree relatives was found in over half of cases. In addition, cardiac risk factor profiles were typical of a population with ACS, with over one half of patients having hypercholesterolemia and hypertension, a third with a history of smoking, and over one fifth with diagnosed diabetes. Previous revascularization had been performed in over a third of the ACS cases.

**Table 1:** Characteristics of 811 white subjects with acute coronary syndrome at baseline

**Characteristic**	Male ACS Cases (N = 550)	Female ACS Cases (N = 261)
Mean age in years (SD)	60.7 (12.5)	63.1 (13.2)
Mean body mass index (SD)	29.1 (5.5)	29.9 (6.9)
Family history of CAD/MI (%)	279 (50.7)	135 (51.7)
Prior myocardial infarction(%)	142 (25.8)	74 (28.4)
Prior revascularization (%)	205 (37.3)	83 (31.8)
Congestive heart failure (%)	23 (4.2)	18 (6.9)
Hypertension (%)	305 (55.5)	182 (69.7)
Diabetes Mellitus (%)	116 (21.1)	77 (29.5)
Hypercholesterolemia (%)	314 (57.1)	162 (62.1)
Postmenopausal (%)	--	189 (68.6)
College graduate (%)	166 (30.2)	40 (15.3)
Smoking <30 days ago (%)	183 (33.3)	85 (32.6)
Alcohol frequency > 1/month (%)	221 (40.2)	38 (14.6)

Continuous variables are shown as mean (SD); categorical variables are number (%)

There were 90 deaths in the cohort, which was followed for a median time of 42.3 months. Risk factor data used in the multivariable models were missing for 2 individuals. Those individuals who died, as compared to survivors, did not differ significantly in sex (P = 0.33) or ACS type (P = 0.22). However, there were marked differences in overall cardiac risk factor profiles, as expected, with deceased patients having relatively advanced age, as well as more extensive cardiovascular comorbidity such as congestive heart failure (Table 2).

**Table 2:** Clinical characteristics of surviving and deceased patients in the follow-up cohort

**Characteristic**	Survivors	Deceased
	(N = 721)	(N = 90)
Mean age in years (SD)‡	60.5 (12.5)	69.5 (11.6)
Mean body mass index (SD)	29.4 (6.0)	28.8 (6.6)
Family history of CAD/MI (%)	374 (52.0)	40 (44.4)
Prior myocardial infarction(%)‡	176 (24.5)	40 (44.4)
Prior revascularization (%)‡	241 (33.5)	47 (52.2)
Congestive heart failure (%)‡	28 (3.9)	13 (14.4)
Hypertension (%)*	423 (58.8)	63 (70.0)
Diabetes Mellitus (%)‡	150 (20.9)	43 (47.8)
Hypercholesterolemia (%)	419 (58.3)	57 (63.3)
Postmenopausal (%)	--	--
College graduate (%)	185 (25.9)	19 (21.6)
Smoking <30 days ago (%)*	249 (34.7)	18 (20.0)
Alcohol frequency > 1/month (%)‡	348 (55.8)	19 (24.4)

Continuous variables are shown as mean (SD); categorical variables are number (%) P < 0.05, *† P < 0.01; ‡ P < 0.001 for comparison between deceased and survivors

A total of 89 variants in 72 genes were genotyped. The overall genotype call rate for these variants was 98.5% (range 95.0–99.8%). Tests of Hardy Weinberg equilibrium revealed that 6 variants violated HWE at the P < 0.05 level, which is not more than expected by chance (P = 0.29), given 89 chi-square tests (Table 2).

The genotype distributions, numbers of deaths by genotypic category, and unadjusted P values for all 89 genetic variables are shown in Table 3. Overall, there were 6 positive associations (P < 0.05). Despite their established association with ACS, none of the 9p21 variants was associated with prognosis. Likewise, the -455 A/G promoter variant in B-fibrinogen, which had been weakly associated with ACS occurrence in our study population, was not associated with post-ACS mortality (P = 0.1). However, the *ACE1 *I/I genotype was associated with a lower survival time (P = 0.04). In addition, the *APOA1 *-75G/A polymorphism was associated with mortality (early death of a single individual with the rare A/A genotype). Survival time was relatively decreased among the 16 individuals with the rare A/A (glutamine homozygous) genotype of the *F7 *Arg353Gln polymorphism (P = 0.01), with 5 deaths in this group. There was also excess mortality among heterozygotes for the *HFE *hemochromatosis-related allele (P = 0.04). In addition, individuals with the A/A genotype (lysine homozygotes) of the *ICAM1 *Lys469Glu missense mutation had an increased mortality risk (P = 0.01). Finally, the strongest statistical association with post-ACS mortality was observed in *IRS1 *arginine homozygotes, owing mainly to 2 early deaths among the 3 individuals with the rare A/A genotype in the Gly972Arg polymorphism; the deceased individuals were two males, aged 71 and 72 years, respectively, both of whom had extensive cardiac risk factor profiles. Heterozygotes had relatively low risk of death compared with homozygotes.

**Table 3:** Kaplan-Meier analysis of mortality post-ACS for 89 genetic variants.

Gene/SNP	Genotype	Total N	Deaths (%)	P	Gene	Genotype	Total N	Deaths (%)	P
**ABCA1**	CC	191	18 (9.4)	0.50	**ICAM1**	AA	270	43 (15.9)	0.01
-477C/T	CT	396	43 (10.9)		Lys469Glu	AG	379	32 (8.4)	
	TT	188	25 (13.3)			GG	145	14 (9.7)	
**ABCA1**	AA	65	7 (10.8)	0.47	**IL1B**	CC	359	35 (9.8)	0.42
Lys219Arg	AG	311	39 (12.5)		-511C/T	CT	311	33 (10.6)	
	GG	416	41 (9.9)			TT	82	12 (14.6)	
**ACE1**	DD	233	28 (12)	0.04	**IL6**	CC	142	16 (11.3)	0.59
I/D	DI	389	35 (9)		-174G/C	CG	386	39 (10.1)	
	II	154	25 (16.2)			GG	277	35 (12.6)	
**ADD1**	GG	456	59 (12.9)	0.23	**IRS1**	AA	3	2 (66.7)	0.001
Gly460Trp	GT	269	25 (9.3)		Gly972Arg	AG	84	4 (4.8)	
	TT	26	2 (7.7)			GG	704	81 (11.5)	
**ADRB2**	CC	266	36 (13.5)	0.19	**ITGA2**	AA	123	17 (13.8)	0.46
Glu27Gln*	CG	358	33 (9.2)		Phe807Phe	AG	394	45 (11.4)	
	GG	146	15 (10.3)			GG	288	28 (9.7)	
**ADRB2**	CC	789	89 (11.3)	0.41	**ITGB3**	CC	20	3 (15)	0.89
Ile164Thr	CT	18	1 (5.6)		Leu33Pro	CT	188	21 (11.2)	
	TT					TT	588	64 (10.9)	
**ADRB2**	AA	128	21 (16.4)	0.09	**LIPC**	CC	506	59 (11.7)	0.79
Gly16Arg	AG	348	36 (10.3)		-514T/C	CT	256	26 (10.2)	
	GG	309	31 (10)			TT	42	5 (11.9)	
**ADRB3**	CC	6	1 (16.7)	0.53	**LPL**	AG	29	3 (10.3)	0.93
Arg64Trp	CT	111	16 (14.4)		Asp9Asn	GG	765	86 (11.2)	
	TT	687	72 (10.5)			GG			
**AGT**	CC	143	19 (13.3)	0.19	**LRP1**	AA	367	37 (10.1)	0.08
Thr235Met	CT	387	48 (12.4)		Thr3261Thr	AG	330	46 (13.9)	
	TT	272	23 (8.5)			GG	85	5 (5.9)	
**AGTR1**	AA	388	48 (12.4)	0.56	**LTA**	AA	394	44 (11.2)	0.52
A1166C	AC	339	34 (10)		A252G	AG	327	38 (11.6)	
	CC	79	8 (10.1)			GG	81	6 (7.4)	
**ALOX5AP**	A	123	12 (9.8)	0.61	**LTA**	AA	80	6 (7.5)	0.51
HAP A	non-A	648	74 (11.4)		Thr26Asn	AC	331	39 (11.8)	
						CC	389	44 (11.3)	
**ALOX5AP**	B	49	3 (6.1)	0.23	**MGP**	AA	308	36 (11.7)	0.81
HAP B	non-B	722	83 (11.5)		Thr83Ala	AG	374	39 (10.4)	
						GG	123	15 (12.2)	
**APOA1**	AA	23	2 (8.7)	0.91	**MGP**	AA	110	13 (11.8)	0.61
C83T	AG	219	25 (11.4)		-7A/G	AG	368	37 (10.1)	
	GG	532	59 (11.1)			GG	328	40 (12.2)	
**APOA1**	AA	1	1 (100)	0.01	**MMP3**	DD	194	20 (10.3)	0.22
-75G/A	AG	10	1 (10)		indel	DI	386	37 (9.6)	
	GG	784	87 (11.1)			II	206	29 (14.1)	
**APOE**	E4/E4	29	1 (3.5)	0.20	**MTHFR**	CC	350	39 (11.1)	0.53
E2-3-4	Non-E4	771	87 (11.3)		Ala222Val	CT	341	40 (11.7)	
						TT	102	8 (7.8)	
**APOE**	GG	194	25 (12.9)	0.42	**MTP**	GG	449	54 (12)	0.13
-219T/G	GT	403	47 (11.7)		-493G/T	GT	297	33 (11.1)	
	TT	206	18 (8.7)			TT	59	2 (3.4)	
**BDKRB2**	CC	263	25 (9.5)	0.40	**MTR**	AA	529	56 (10.6)	0.15
-58C/T	CT	394	50 (12.7)		Asp919Gly	AG	239	32 (13.4)	
	TT	145	14 (9.7)			GG	34	1 (2.9)	
**CCL11**	CC	539	64 (11.9)	0.76	**NPPA**	CC	22	2 (9.1)	0.65
Thr23Ala	CT	239	24 (10)		Ter29ArgArg	CT	190	18 (9.5)	
	TT	12	1 (8.3)			TT	583	70 (12)	
**CCR2**	AA	7	1 (14.3)	0.96	**OLR1**	CC	649	75 (11.6)	0.27
Val64Ile	AG	116	12 (10.3)		Lys167Asn	CG	146	12 (8.2)	
	GG	681	77 (11.3)			GG	8	2 (25)	
**CCR5**	II	631	72 (11.4)	0.51	*P22*-**PHOX**	CC	347	26 (7.5)	0.05
Indel	ID	162	18 (11.1)		His72Tyr**	CT	271	36 (13.3)	
	DD	12	0 (0)			TT	121	15 (12.4)	
**CD14**	CC	204	25 (12.3)	0.22	**PAI1**	DD	249	32 (12.9)	0.33
-159C/T	CT	395	47 (11.9)		indel	DI	398	38 (9.6)	
	TT	204	16 (7.8)			II	159	20 (12.6)	
**CETP**	AA	168	13 (7.7)	0.13	**PECAM1**	CC	187	21 (11.2)	0.86
intron1 G/A	AG	387	51 (13.2)		Leu125Val	CG	395	42 (10.6)	
	GG	250	25 (10)			GG	222	27 (12.2)	
**CETP**	AA	205	18 (8.8)	0.39	**PECAM1**	AA	200	25 (12.5)	0.82
-629C/A	AC	400	50 (12.5)		Ser563Asn	AG	386	42 (10.9)	
	CC	197	21 (10.7)			GG	214	22 (10.3)	
**COMT**	AA	231	17 (7.4)	0.06	**PON1**	AA	396	36 (9.1)	0.09
Val158Met	AG	358	43 (12)		Gln192Arg	AG	337	47 (14)	
	GG	187	27 (14.4)			GG	66	6 (9.1)	
**CX3CR1**	CC	410	43 (10.5)	0.54	**PON2**	CC	464	56 (12.1)	0.43
Ile249Val	CT	336	40 (11.9)		Cys311Ser	CG	298	28 (9.4)	
	TT	56	4 (7.1)			GG	40	3 (7.5)	
**CX3CR1**	AA	18	1 (5.6)	0.73	**PPARG**	CC	637	73 (11.5)	0.32
Thr280Met	AG	223	26 (11.7)		Ala12Pro	CG	159	15 (9.4)	
	GG	565	63 (11.2)			GG	8	2 (25)	
**CYP11B2**	CC	163	18 (11)	0.84	**PTGS2**	CC	15	2 (13.3)	0.94
-344T/C	CT	352	42 (11.9)		-765G/C	CG	202	22 (10.9)	
	TT	275	29 (10.6)			GG	576	65 (11.3)	
**CYP2C9**	AA	708	79 (11.2)	0.89	**RECQL2**	CC	66	11 (16.7)	0.18
Leu359Ile	AC	56	6 (10.7)		Arg1367Cys	CT	326	39 (12)	
	CC					TT	412	39 (9.5)	
**CYP2C9**	CC	589	63 (10.7)	0.83	**SELE**	CC	658	71 (10.8)	0.43
Cys144Arg**	CT	147	15 (10.2)		Leu554Phe	CT	137	18 (13.1)	
	TT					TT	7	0 (0)	
**ENPP1**	AA	600	64 (10.7)	0.75	**SELE**	AA	740	82 (11.1)	0.89
Gln121Lys	AC	192	24 (12.5)		Ser128Arg	AC	63	7 (11.1)	
	CC	15	2 (13.3)			CC	2	0 (0)	
**ESR1**	CC	145	21 (14.5)	0.32	**SELP**	AA	646	71 (11)	0.48
-401T/C	CT	421	41 (9.7)		Thr715Pro	AC	150	19 (12.7)	
	TT	239	27 (11.3)			CC	9	0 (0)	
**F12**	CC	459	51 (11.1)	0.73	**TFPI**	AA			
46C/T	CT	283	32 (11.3)		Val264Met	AG	32	4 (12.5)	0.71
	TT	49	7 (14.3)			GG	758	84 (11.1)	
**F13A1**	GG	443	44 (9.9)	0.23	**THBD**	AA			
Val34Leu	GT	296	39 (13.2)		-33G/A	AG	3	1 (33.3)	0.23
	TT	41	7 (17.1)			GG	801	89 (11.1)	
**F2**	AA	1	0 (0)	0.93	**THBD**	AA	11	2 (18.2)	0.46
G20210A*	AG	23	2 (8.7)		Ala25Thr	AG	794	87 (11)	
	GG	783	88 (11.2)			GG			
**F5**	AA	1	0 (0)	0.26	**THBD**	CC	531	64 (12.1)	0.52
Arg506Gln	AG	36	1 (2.8)		Ala455Val	CT	237	22 (9.3)	
	GG	769	89 (11.6)			TT	24	2 (8.3)	
**F7**	AA	16	5 (31.3)	0.01	**THBS1**	AA	614	66 (10.8)	0.65
Arg353Gln	AG	148	19 (12.8)		Asn700Ser	AG	177	23 (13)	
	GG	629	63 (10)			GG	14	1 (7.1)	
**FGB**	AA	24	0 (0)	0.10	**THBS2**	GG	74	10 (13.5)	0.65
-455A/G	AG	247	23 (9.3)		3'UTR T/G*	GT	250	25 (10)	
	GG	533	67 (12.6)			TT	466	53 (11.4)	
**GJA4**	CC	401	36 (9)	0.14	**THBS4**	CC	49	6 (12.2)	0.53
C1019T	CT	313	38 (12.1)		Ala387Pro	CG	268	34 (12.7)	
	TT	78	13 (16.7)			GG	486	49 (10.1)	
**GP1BA**	CC	13	1 (7.7)	0.87	**THPO**	AA	187	25 (13.4)	0.52
-5T/C	CT	168	21 (12.5)		A5713G	AG	374	40 (10.7)	
	TT	597	66 (11.1)			GG	241	24 (10)	
**GRL**	AA	756	83 (11)	0.68	**TLR4**	AA	702	85 (12.1)	0.10
Asn363Ser	AG	47	6 (12.8)		Gly299Asp	AG	88	4 (4.6)	
	GG					GG	1	0 (0)	
**HFE**	AA	3	0 (0)	0.04	**TNF**	AA	24	4 (16.7)	0.43
Cys282Tyr	AG	96	18 (18.8)		-308G/A	AG	784	86 (11)	
	GG	703	70 (10)			GG			
**HTR2A**	CC	286	25 (8.7)	0.13	**TNFRSF1A**	AA	17	0 (0)	0.29
Ser102Ser	CT	363	49 (13.5)		Arg92Gln	AG	189	19 (10.1)	
	TT	134	16 (11.9)			GG	597	71 (11.9)	
**9p21**	GG	114	15 (13.2)	0.33	**9p21**	AA	175	23 (13.1)	0.28
rs10116277	GT	365	41 (11.2)		Rs10757274	AG	375	44 (11.7)	
	TT	243	20 (8.2)			GG	226	19 (8.4)	
**9p21**	CC	118	11 (9.3)	0.24					
Rs1333040	CT	332	44 (13.3)		**9p21**	AA	146	17 (11.6)	0.35
	TT	306	28 (9.2)		Rs2383206	AG	375	46 (12.3)	
**9p21**	AA	78	8 (10.3)	0.25		GG	249	21 (8.4)	
Rs2383207**	AG	366	46 (12.6)						
	GG	268	22 (8.2)						

*HWE deviation (P < 0.05); **HWE deviation (P < 0.01)

Of the 6 positive associations described above, 2 remained significant following Cox regression adjustment for traditional cardiac risk factors including age, sex, hypertension, diabetes, congestive heart failure, and ACS type, as well as treatment-related factors. Of these covariates, age (P < 0.0001), diabetes (P < 0.0001), and lack of revascularization (P < 0.01) had the most significant associations with mortality when modeled independent of genotype. The G allele of *IRS1 *was related to longer survival, with adjusted hazard ratios of 0.08 (0.02, 0.84) for A/G, and 0.23 (0.05, 0.99) for G/G, respectively. In addition, the *ICAM1 *association remained significant, only for A/G heterozygotes (0.53; 95% CI: 0.33, 0.84), with the reference genotype being A/A.

**Figure 1:**
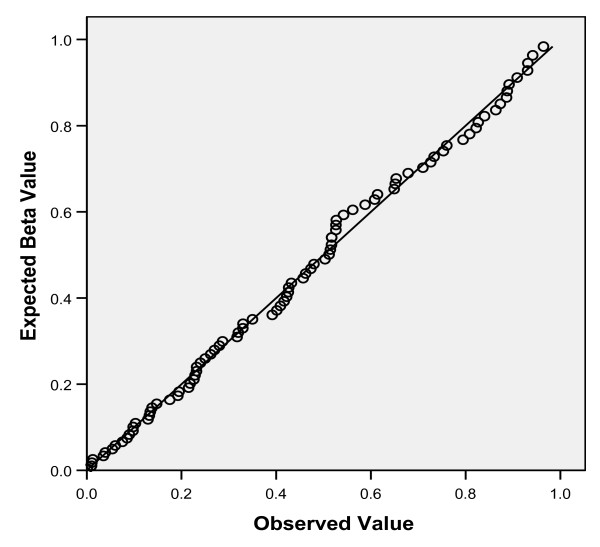
**Q-Q plot of Kaplan-Meier P values for 89 genetic variants**. Observed log-rank P values for individual Kaplan-Meier analyses are plotted against random expectations under the null hypothesis of no effect.

**Figure 2:**
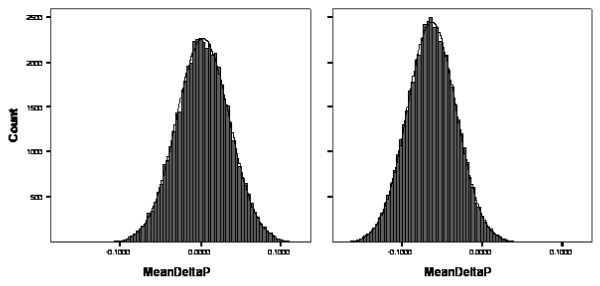
**Mean P value differences (observed-random) in 50,000 simulations**. The right panel shows a skewed distribution of simulated random minus actual Kaplan-Meier log-rank P values (N = 89), indicating that observed P values collectively were lower than expected. With the removal of 16 positive associations (P < 0.1), the remaining genetic variables closely approximated a random distribution, as shown in the left panel.

In order to further explore the data for potential weak associations not quite meeting the P < 0.05 significance threshold due to suboptimal power, we performed a simulation analysis as described above, to supplement visual inspection of the Q-Q plot (Figure 1), which was not precisely linear. The mean difference that we observed was -0.065 for all 89 genetic variables (Figure 2), indicating that some P values were lower than expected by chance alone. In addition to the 6 associations described above, 10 additional genetic variants had P values of 0.1 or lower. Upon removal of these 16 positive associations (P < 0.1), the mean difference converged to zero in the remaining 73 genetic variables (Figure 2), suggesting no probable association with mortality.

## Discussion

Of the 89 genetic variants tested, we found strong statistical evidence of an association for only one, the *IRS1 *Gly972Arg polymorphism, although it was based upon a small number of deaths in individuals who would already have been considered at high risk for mortality. Thus, we can not unequivocally implicate any of the genetic variants included in this study as a prognostic factor following ACS, including those in the 9p21 region. The possible explanations for our essentially null findings include inadequate power for survival analysis, given that mortality following ACS occurred in only 11.1% of our case sample, or that the genetic variants included in our study have no impact on post-MI prognosis.

With respect to statistical power, we have presented evidence that at least 73 of the gene variants seemed identical to random variables, and therefore, our data provide no support for the hypothesis that testing these particular variants in larger samples would likely yield positive results. It is unlikely that mortality in direct relation to any of these gene variants is a common occurrence following ACS. Furthermore, as shown in Table 1, conventional cardiac risk factors such as advanced age and diabetes appear to be dominant factors in determining prognosis, and therefore, it may be challenging to demonstrate an independent genetic effect on mortality without sample sizes far in excess of 800 patients, particularly if genetic or pharmacogenetic interactions are explored.

However, given the limitations of our sample size, we were unable to exclude modest effects in association with the remaining 16 gene variants, 6 of which had nominally statistically significant associations with post-ACS mortality. Of these, only 2 remained significant after adjustment for traditional cardiac risk factors, with *ACE1*, *APOA1*, *F7, and HFE *having no independent association. As might have been expected, known confounding variables such as age, sex, and comorbidity proved to be important predictors of mortality in the Cox regression models. In addition, *APOA1 *was related to a small number of deaths, and the association of the I/I genotype of *ACE1 *would seem paradoxical, given that the D/D genotype has been postulated as a cardiac risk factor in most studies.

As with *ACE1*, the association of the *F7 *glutamine allele would also have to have been considered paradoxical, *a priori*, given that this allele was originally reported to be protective against the occurrence of myocardial infarction, and it correlates with lower *F7 *activity and hence lesser coagulability. However, it was the relatively rare A/A genotype of *F7 *that conferred the greatest risk of mortality in our population of ACS patients, which is consistent with the hypothesis that rare variants may have relatively large genetic effects. Interestingly, all 5 deceased individuals with the A/A genotype had NSTEMI, which is improbable (P = 0.005) given that only 35% of the cases had this manifestation of ACS, and 4 of 5 had either prior MI (N = 3) or a history of revascularization (N = 1). Thus, further study of the *F7 *allele is warranted, especially in NSTEMI patients.

In contrast, it is possible to postulate a plausible cardiovascular risk mechanism for the hemochromatosis-associated A allele. Heterozygotes for the A allele, on average, have relatively increased total body iron stores.[19,20] In addition, some data indicate that iron excess may lead to free radical formation and thereby confer risk of myocardial infarction.[21] However, a limitation of our study is a lack of phenotypic data on serum iron stores (e.g., ferritin or serum iron levels). In addition, the existing literature on *HFE *does not show a consistent association with cardiac disease, and the allele was not even marginally associated with the occurrence of ACS in our study population.[5]

In judging the likely validity of the remaining 2 genetic associations that remained statistically significant despite extensive adjustment for comorbidity and treatment variables, one must consider the magnitude of the risk, the numbers of deaths observed, as well as the relationship of the genotypic risk pattern in the context of the functional biology of each particular gene.[22]

The *ICAM1 *Lys469Glu association was related to relatively increased mortality (15.9%) among individuals with the A/A genotype (lysine homozygotes). This is higher than the overall 11.1% mortality rate in the cohort, and there were 43 deaths among individuals with this frequent (34.0%) genotypic class. However, it is the 469Glu allele that is postulated to have deleterious pro-inflammatory effects,[23] and therefore, no biological mechanism for this association is immediately obvious.

The strongest genetic association was detected for the rare A allele (coding for arginine) of the *IRS1 *Gly972Arg polymorphism. Even a Bonferroni-corrected P value for this association had borderline (P < 0.1) study-wide significance. IRS1 is activated as an intracellular signal transducer, in adipocytes and skeletal muscle cells, when its tyrosine residues are phosphorylated by the insulin-bound insulin receptor, the function of which is inhibited *in vitro *by the Arg972 residue, leading to decreased glucose uptake.[24,25] Baroni *et al *(1999) reported the A allele as a risk factor for coronary artery disease (18.9% vs. 6.8%),[25] and there have been inconsistent reports of this polymorphism being more prevalent among diabetics,[26,27] perhaps causing defective phosphatidylinositol 3-kinase interaction, peripheral insulin resistance, and impaired insulin secretion. In addition, Harrap *et al *reported a subthreshold linkage peak containing *IRS1 *in a genome-wide scan of 61 sibling pairs with acute coronary syndrome, adding to interest in the *IRS1 *locus as a cardiac candidate gene.[28] However, IRS1 was not associated with the primary occurrence of ACS in our previous case-control analysis.[5] Moreover, due to the rarity of arginine homozygous genotype, the association in our study population was largely based on the deaths of 2 individuals among the 3 with the A/A genotype. Accordingly, further prognostic study of this rare genotype is warranted in larger populations of ACS patients, particularly those with diabetes.

In 3 of the 6 nominally significant bivariate associations described above (*ACE*, *F7*, *ICAM1*), the allele hypothesized to be protective against ACS occurrence was associated with early death following ACS, creating an apparent paradox. These may be false positive associations. However, such apparently paradoxical, but well-validated, associations have been observed in prognostic studies (e.g., the association between active smoking and lower mortality following ACS).[29] One theoretical explanation could be survivorship bias, meaning that most individuals with the genetic risk factor never survive the initial cardiac event, and that the remaining individuals are those with the best prognosis. Although potentially interesting, this hypothesis is difficult to investigate except prospectively, with ascertainment prior to the initial cardiac event.

## Conclusion

In summary, our study has provided no uneqivocal support for the hypothesis that any of 89 genetic variants in 72 genetic loci contribute to death following ACS. However, in the context of multiple genetic comparisons, and necessarily modest numbers of deaths in a cohort of 811 individuals with ACS, it is challenging to interpret P values of borderline study-wide significance. Thus, we can not exclude relatively modest survival effects in the 16 gene variants with P < 0.1 (Table 3). Further study of these variants would be warranted, and meta-analysis would augment power to detect weak effects. In addition, given the recent success of the whole genome association approach in the identification of 9p21 and other promising cardiovascular candidate genes, it is plausible that this approach may have similar success in identifying genetic risk factors for prognosis following ACS.

## Competing interests

Dr. Krumholz discloses that he has research contracts with the Colorado Foundation for Medical Care and the American College of Cardiology, serves on the advisory boards for Amgen, Alere and United Healthcare, is a subject matter expert for VHA, Inc., and is Editor-in-Chief of Journal Watch Cardiology of the Massachusetts Medical Society. The other authors have no conflicts of interest to report.

## Authors' contributions

TMM performed genotyping and data analysis, and collaborated with JAS and HMK (who assembled the patient cohort) on study design and manuscript preparation. LX provided statistical analysis. PL and BK participated in genotyping, and genetic data analysis. All authors read and approved the final manuscript.

## Pre-publication history

The pre-publication history for this paper can be accessed here:


